# Perovskite Light-Emitting Devices Based on Solid-State Diffusion In Situ Dynamic Thermal Crystallization

**DOI:** 10.3390/mi14112084

**Published:** 2023-11-11

**Authors:** Chen Chen, Yanni Zhu, Kainan Dou, Chuang Liu, Chao Yu, Sihang Ji, Jin Wang

**Affiliations:** 1 College of Information Technology, Jilin Normal University, Siping 136000, China; 18843415834@163.com (Y.Z.); hnl008@163.com (K.D.); haythamekenway@126.com (C.L.); sihangji@163.com (S.J.); 2 State Key Laboratory of Integrated Optoelectronics, College of Electronic Science and Engineering, Jilin University, Changchun 130015, China; chaoyu19@mails.jlu.edu.cn

**Keywords:** light-emitting devices, in situ dynamic thermal crystallization, solid-state diffusion

## Abstract

Due to the excellent photonic and electrical properties of metal halide perovskite materials, perovskite light-emitting devices have the potential to replace OLED devices as the next-generation of commercial light-emitting devices. In this article, we controlled the surface morphology of PbBr_2_ using an in situ dynamic thermal crystallization process, which increased the specific surface area of the films and promoted the solid-state diffusion rate. The CsPbBr_3_ PeLEDs prepared using this method achieved a maximum current efficiency of 7.1 cd/A at the voltage of 5 V, which was 200% higher than devices prepared using traditional spin-coating processes. These results proved that the in situ thermal dynamic crystallization process effectively improved the film quality of perovskite materials.

## 1. Introduction

Due to the specific characteristics such as light stability and thermal stability [1,2,3,4,5], all-inorganic perovskite materials are expected to replace organic/inorganic hybrid perovskite materials as the mainstream of perovskite optoelectronic devices in the future [1,6,7,8,9,10]. However, rapid solvent evaporation during solution processing tends to produce nonuniform film morphologies, whereas low CsBr solubility in organic solvents severely limits the practical application of PeLEDs [9,11,12,13]. At the same time, it also directly affects the repeatability of the preparation process of PeLEDs [14,15,16]. The tight, void-free interfacial contact between the perovskite layer and the transport layer helps to minimize nonradiative composite at the interface [17,18,19,20].

In 2018, Professor Qunwei Tang’s team from Jinan University reported a method to control the film morphology by adjusting the spin-coating times of CsBr on a PbBr_2_ substrate. Although the PCE of 9.72% was obtained after optimization, it actually still required the assistance of a mesoporous layer to facilitate the diffusion of the CsBr precursor [17]. A mesoporous structure is useful for perovskite films prepared based on the two-step method; however, for LEDs, a planar structure is a better choice [21]. In 2015, Professor Nam-Gyu Park’s team from Sungkyunkwan University first reported a planar CH_3_NH_3_PbI_3_ perovskite solar cell prepared by substrate preheating technology. The researchers believed that the morphology of PbI_2_ played a crucial role in the performance of the devices [22]. Compared with the substrates without preheating treatment, the increase in PbI_2_ crystallinity made possible by the preheating technology is the main reason for the increase in the photocurrent and voltage of the device. Therefore, it is expected that in situ dynamic thermal crystallization will play a crucial role in regulating the morphology of precursor films. In current research on using spin-coating technology to measure in situ dynamic thermal crystallization temperature, the substrate is often preheated to a certain temperature and then placed on a spin-coating device for film preparation. However, in current studies on the thermal dynamic crystallization, the substrates were often preheated to a certain temperature and then placed on spin-coating equipment for preparation. During the spin-coating process, the temperatures of the substrates continued to decrease, which affected the accuracy of the experimental results. In the Support information, we validated this viewpoint through experiments. One of the major drawbacks of the traditional two-step solution deposition route is that for CsPbBr_3_, the perovskite always suffers from low phase purity and poor morphology. Generally, in the conventional one-step and two-step solution-based processes, enhancing the device efficiency of CsPbBr_3_ is difficult due to the large difference in the concentration between the CsBr and PbBr_2_ solutions and the formation of mixed phases. Also, the phase conversion of CsPbBr_3_ to Cs_2_PbBr_5_ and Cs_4_PbBr_6_ substantially reduces the efficiency of the devices [18]. In order to simulate the cooling process of the preheated substrate during spin coating and verify the necessity of in situ thermal-assisted technology, we designed a cooling rate experiment at room temperature for the preheated substrate. When the room temperature environment is 23 °C, we heat the substrate to 60 °C and then remove it and place it on the desktop. We found that it only takes 30 s for the in situ thermal-assisted crystallization temperature to decrease to half of the initial temperature (preheating temperature), and within one minute, the in situ thermal-assisted crystallization temperature will decrease to room temperature. Therefore, it is necessary to study the effect of in situ thermal-assisted crystallization temperature on the film formation and crystallization characteristics of perovskite films using in situ thermal-assisted crystallization technology. (Refer to the Supplementary Information Figure S1.)

In this article, we deposited CsBr by vacuum thermal evaporation on the surface of PbBr_2_ films prepared by spin coating to prepare CsPbBr_3_ films. Based on this, we controlled the surface morphology of PbBr_2_ films through in situ dynamic thermal crystallization to obtain CsPbBr_3_ films with better surface coverage and fewer defects. We also prepared PeLEDs devices based on this process.

## 2. Experimental Section

Materials Preparation. DMF (99.9%), IPA, cesium bromide (CsBr, 99.99%), lead bromide (PbBr_2_, 99.99%) and the ITO substrates were purchased from Advanced Election Technology Co., Ltd. (Taipei, China). PEDOT: PSS CLEVIOS P VP Al 4083 (1.3–1.7 wt% solution on water), 1,3,5-tris(1-phenyl-1H-benzimidazol-2-yl) benzene Synonym (TPBI, 99.5%), and 8-hydroxyquinolinolato-lithium (Liq, 99.5%) were purchased from Xi’an Yuri Solar Co., Ltd. (Xi’an, China). Aluminum (Al) was purchased from Beijing Dream Material Technology Co., Ltd. (Beijing, China).

### Device Fabrication

**Figure 1:**
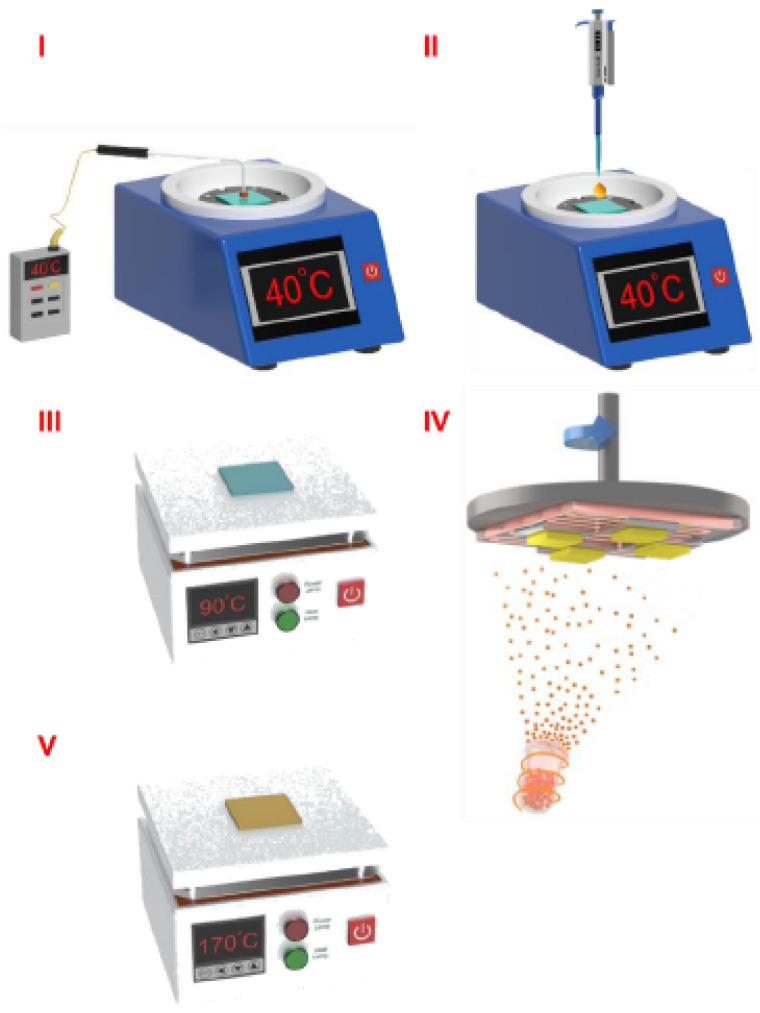
The flow chart of the in situ dynamic thermal crystallization for the two-step solid-solid diffusion method.

Figure 1 shows the flow chart of the in situ dynamic thermal crystallization for the two-step solid-solid diffusion method. Before starting the spin-coating process, we preset the in situ dynamic thermal crystallization temperatures on the temperature-controlled spin-coating equipment in the range of 25 to 80 °C. The ambient temperature of the glove box was maintained in the range of 24 to 26 °C by the glove box air conditioner. In this manuscript, the room temperature (RT) represented 25 °C. The specific deposition process was as follows:

- When the temperature has risen to the preset temperature, the substrate was placed on a suction cup, and the calibrated thermocouple was used to directly contact the substrate to measure its temperature.
- We added the precursor solution of PbBr_2_ to the surface of ITO/PEDOT:PSS quickly. The spin-coating speed of the PbBr_2_ precursor solution was 5000 rpm, and spin-coating acceleration was 2500 rpm/s. The spin-coating time was 30 s.
- The PbBr_2_ film was annealed at 90 °C for 30 min.
- Then, we transferred the film to the vapor thermal deposition system to deposit the CsBr.
- After preparation, the film was annealed at 170 °C for 10 min.

## 3. Result and Discussion

**Figure 2:**
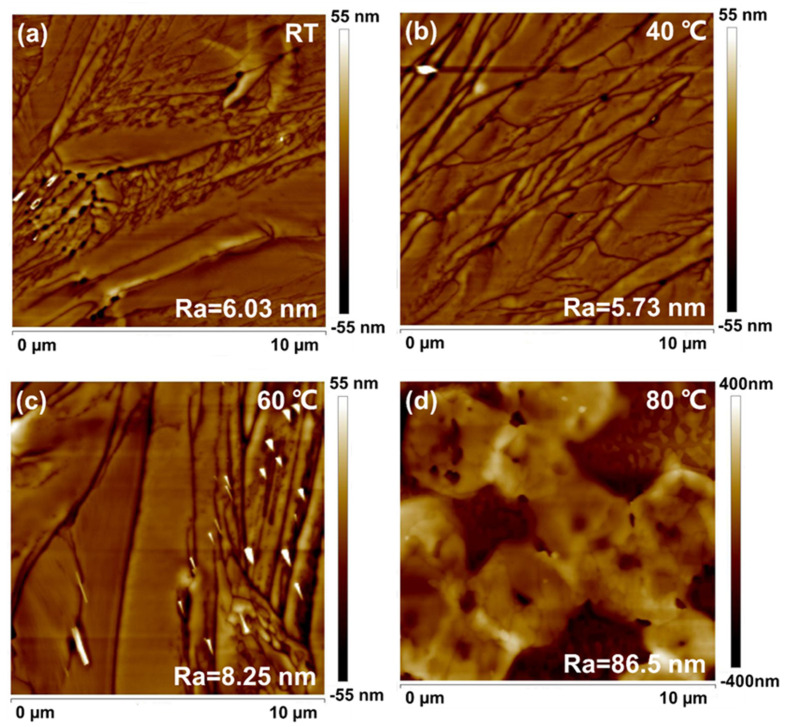
(**a**–**d**) The AFM of PbBr_2_ films prepared at different in situ thermally dynamic crystallization temperatures after annealing.

The AFM of PbBr_2_ films prepared at different in situ thermally dynamic crystallization temperatures after annealing is shown in Figure 2a–d. When the temperature was increased from RT to 40 °C, the surface morphology of PbBr_2_ was the flattest, with Ra measuring only 5.73 nm. As the temperature increased to 60 °C, the surface roughness of the PbBr_2_ film increased to 8.25 nm. When the temperature increased to 80 °C, the surface roughness of the film jumped to 86.5 nm. In Figure 2b, it could be seen that the PbBr_2_ films exhibited a dense and non-porous dendritic morphology, which could be the reason for the smoother surface. 

The step profiler was used to assess the average thickness of films comprising PbBr_2_ deposited at RT and temperatures of 40 °C, 60 °C, and 80 °C through in situ thermally dynamic crystallization. The findings highlight that the thickness of the PbBr_2_ films escalated as the temperature increased, ranging between 38 and 150 nm. The results of this study reveal that the increased temperature results in a competition between the PbBr_2_ crystallization rate and DMF evaporation rate. The results of this study reveal that the increased temperature results in a competition between the PbBr_2_ crystallization rate and DMF evaporation rate. The PbBr_2′_s crystallization rate, in particular, is significantly higher than the solvent’s evaporation rate. 

Since the film thicknesses of PbBr_2_ films prepared at different in situ thermally dynamic crystallization temperatures were different, the thicknesses required for the subsequent reaction of CsBr were calculated by taking the two precursor materials, CsBr and PbBr_2_, required for the synthesis of CsPbBr_3_ chalcogenides films at a molar ratio of 1:1. Equation (1) shows the corresponding mass ratio when the molar ratio of the two is 1:1:

(1) $$\frac{M_{\mathrm{CsBr}}}{M_{{\mathrm{CsBr}}_{2}}}=\frac{m_{\mathrm{CsBr}}}{m_{{\mathrm{CsBr}}_{2}}}$$

(2) m = ρ × V = ρ × S × h

Here, the relative molecular mass is represented by M, mass is represented by m, density is represented by P, contact area is represented by S, and thickness is represented by H. CsBr and PbBr_2_ were the two precursor materials that react with the same area. Therefore, using Equations (1) and (2), we had calculated the thickness of the corresponding CsBr. 

**Figure 3:**
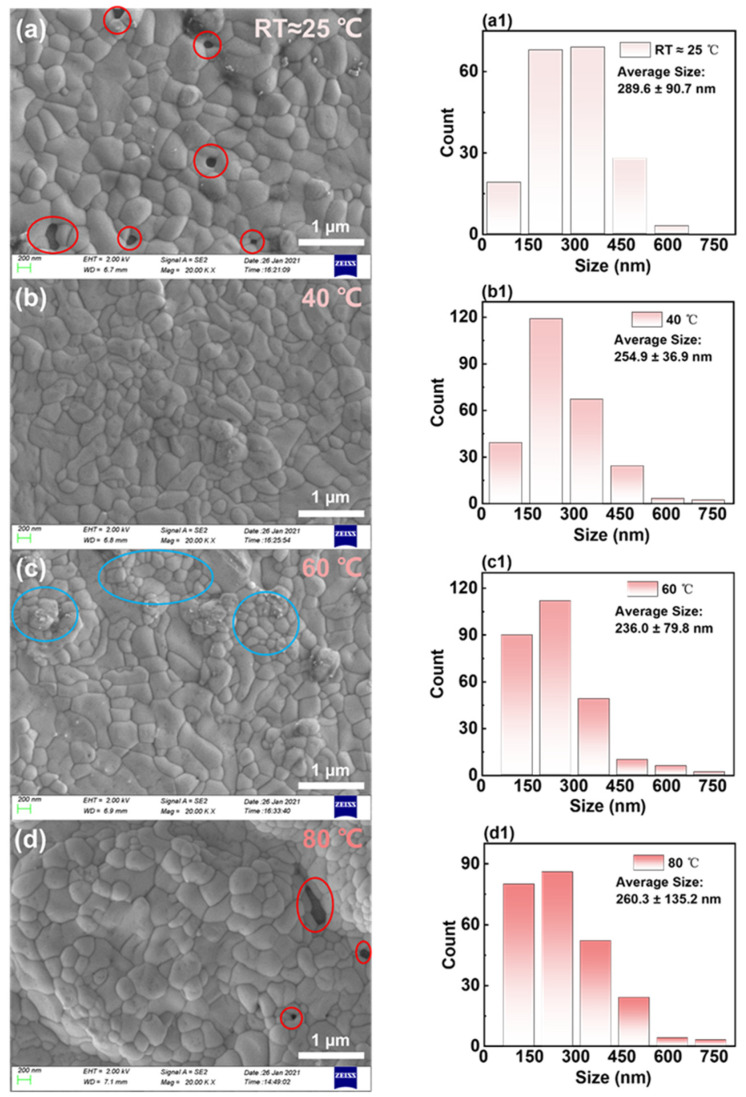
(**a**–**d**) The SEM images of CsPbBr_3_ films prepared based on PbBr_2_ films deposited at RT ≈ 25 °C, 40 °C, 60 °C, and 80 °C in situ heat dynamic crystallization temperatures, respectively. The corresponding grain size distribution and average grain size are shown in (**a1**–**d1**).

To explore the effects of surface morphology of PbBr_2_ films on the creation of CsPbBr_3_ films, the surface morphology of CsPbBr_3_ films was analyzed. The SEM images of CsPbBr_3_ films created using PbBr_2_ films deposited at varying temperatures are displayed in Figure 3a–d with the corresponding grain size distribution and average grain size shown in Figure 3a1–d1. From the average grain sizes, it was observed that as the in situ thermally dynamic crystallization temperatures increased from 25 to 40 °C, the average grain sizes of the CsPbBr_3_ films decreased from 289 to 254 nm. Furthermore, as the in situ thermally dynamic crystallization temperature was further increased to 60 °C, the average grain size of CsPbBr_3_ continued to decrease to 236 nm. The diminishment in size was linked to the conversion rate between PbBr_2_ and CsPbBr_3_, whereby an elongated conversion reaction time resulted in bigger crystals [22]. 

Similar to single crystals, achieving larger sizes often required several days [23,24]. Therefore, when the thickness of PbBr_2_ films was thin, a longer reaction time was needed, resulting in an increase in grain size. Meanwhile, it is noteworthy that under the thermal conditions of in situ dynamic crystallization at room temperature, as illustrated in Figure 3a, clear voids were observed in the areas highlighted by red circles, which were attributable to grain enlargement and their heterogeneous distribution [25]. 

Research indicated that an optimal perovskite grain size could mitigate surface defects in films while also curbing nonradiative charge carrier recombination [26]. At a temperature of 60 °C, the grains on the surface of the CsPbBr_3_ film are noticeably clustered, as depicted in Figure 3c at the blue circle. This phenomenon is attributable to the grains being relatively small and there being an inadequate reaction. When the thermal crystallization temperature was increased to 80 °C in situ, the PbBr_2_ film became thicker, as shown in Figure 3d. However, due to the significant roughness of the surface morphology grains, the grain size distribution became severely inhomogeneous (with a variance of ±132 nm). As a result, its grain size increased statistically instead. Figure 3d also reveals apparent holes at the position shown by the red circle.

Although the SEM image does not accurately represent the film’s morphology, its light and dark variations reveal the sample surface’s relative height. At a thermally assisted crystallization temperature of 40 °C, as demonstrated in Figure 3b, the SEM image’s light and dark variations are comparatively lower than those of other temperatures. Therefore, besides having a higher surface coverage and more uniformly sized grains, CsPbBr_3_ films may also have flatter surfaces. Consequently, we utilized a 3D optical profiler to evaluate the surface roughness of CsPbBr_3_ films derived from PbBr_2_ films deposited at various in situ thermally assisted crystallization temperatures.

**Figure 4:**
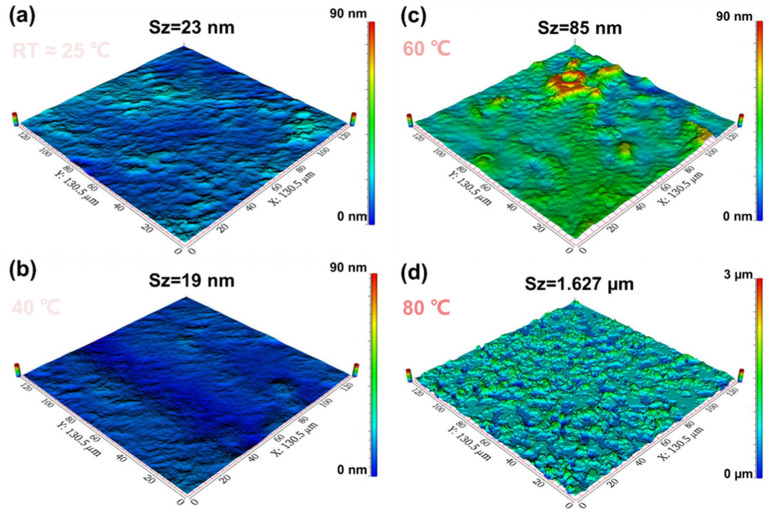
(**a**–**d**) The optical three-dimensional surface profiles of CsPbBr_3_ films prepared based on PbBr_2_ films deposited at RT ≈ 25 °C, 40 °C, 60 °C, and 80 °C in situ dynamic thermal crystallization temperatures, respectively.

The optical 3D surface profiles of CsPbBr_3_ films, which were synthesized from PbBr_2_ films at various in situ dynamic thermal crystallization temperatures, are depicted in Figure 4a–d. The film surface topography’s lowest point is located at zero in all cases. The data illustrate that as the in situ thermally dynamic crystallization temperature rises from room temperature to 40 °C, the CsPbBr_3_ film demonstrated a surface roughness Sa of only 2 nm. Moreover, it can be deduced from Figure 4 that the PbBr_2_ film possessed the smoothest surface, thus substantiating our prior observation that the film produced at 40 °C exhibited better coverage and flatness. When the in situ thermal crystallization temperature increases from 40 to 60 °C, the maximum height of the CsPbBr_3_ film surface (Sz) increases to 85 nm. However, as shown in Figure 4c, the surface morphology of the CsPbBr_3_ film remained relatively flat at a scale of 13.5 μm^2^ with only certain areas exhibiting a bumpy height. This was directly related to the clustering of the grains shown in Figure 4b. The film clustering seen in Figure 4d corresponds to the morphology of PbBr_2_ prepared at an in situ thermally dynamic crystallization temperature of 80 °C. Table 1 displayed the surface roughness test outcomes for CsPbBr_3_ films made using PbBr_2_ films deposited at RT, 40 °C, 60 °C, and 80 °C in situ dynamic thermal crystallization temperatures.

Based on the data presented in Figure 4 and Table 1, it is evident that the CsPbBr_3_ films, produced through in situ heat-dynamic crystallization of PbBr_2_ at 40 °C, possess the flattest surface roughness and the smallest relative height standard deviation.

In summary, the CsPbBr_3_ films produced using the two-step method exhibit uniform grain size and smoother surface morphology attributable to the high flatness and good crystallinity of the PbBr_2_ film that undergoes low-temperature in situ heat-dynamic crystallization process at 40 °C. Theoretically, the CsPbBr_3_ films could display a higher surface roughness than the PbBr_2_ film produced by the in situ heat-dynamic crystallization process. Thus, the growth of CsPbBr_3_ films on these substrates yields a consistently sized grain and smoother surface morphology, ultimately enabling the development of superior chalcogenide optoelectronic devices.

**Figure 5:**
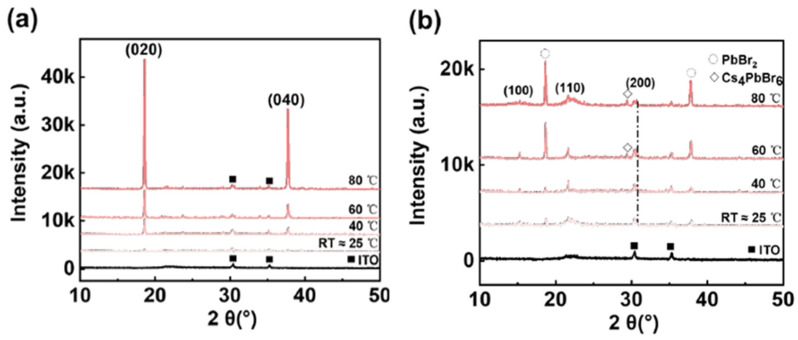
(**a**,**b**) The XRD patterns of PbBr_2_ films and CsPbBr_3_ films prepared based on PbBr_2_ films deposited at RT ≈ 25 °C, 40 °C, 60 °C, and 80 °C in situ dynamic thermal crystallization temperatures, respectively.

To gain a deeper understanding, we analyzed the PbBr_2_ film structure employing an in situ heat-dynamic crystallization process. Figure 5a,b illustrate these processes. X-ray diffraction (XRD) characterization was conducted on PbBr2 thin films generated at varying in situ dynamic thermal crystallization temperatures as well as annealed CsPbBr_3_ film samples, all of which were prepared on ITO/PEDOT: PSS substrates.

In Figure 5a, increasing the temperature of in situ thermally dynamic crystallization reveals strong diffraction peaks at the (020) and (040) crystal planes of PbBr_2_ (at 2θ of 18.6° and 37.5°, respectively). Table 2 shown the width of the half-peak narrows with increasing temperature, indicating a deceleration in the conversion of PbBr_2_ to CsPbBr_3_. This corresponded to the observation shown in Figure 3a–c, where the grain size decreases as the temperature of in situ thermally dynamic crystallization increases. Additionally, Figure 5b demonstrates that the residual PbBr_2_ in the CsPbBr_3_ films prepared via the two-step method exhibits a general increasing trend with the rise of the in situ thermally dynamic crystallization temperature. Nevertheless, the amount of residual PbBr_2_ is at its minimum at 40 °C. Based on our observations of the surface morphology of the PbBr_2_ films, we infer that their dense and uniform dendritic structure at a temperature of 40 °C could account for a larger specific surface area for CsBr to react with, thereby minimizing the amount of residual PbBr_2_. Increasing the in situ thermally dynamic crystallization temperature above 60 °C resulted in a notable peak of Cs_4_PbBr_6_ at a 2θ of 28.6°—clearly indicating the presence of a chemical reaction. This could be attributed to the reaction between the lower PbBr_2_ and CsBr to form CsPbBr_3_, which is followed by continuous reaction with the unreacted CsBr, which results in the formation of Cs_4_PbBr_6_.

The CsPbBr_3_ films, prepared through in situ thermally dynamic crystallization at 40 °C, exhibit the strongest diffraction peaks on the (110) crystal plane. The light-emitting performances of the previous chapters suggests that the 40 °C crystallization temperature is a more suitable option for the preparation of electroluminescent devices.

**Figure 6:**
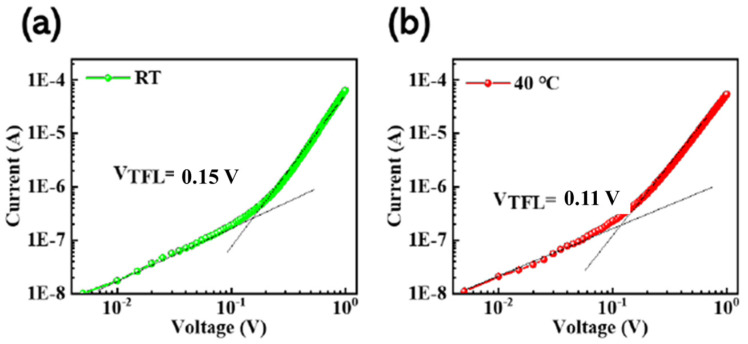
(**a**) and (**b**) depict the in situ dynamic thermal crystallization of CsPbBr_3_ at RT and 40 °C, respectively. Additionally, a diagram of the single-hole carrier device SCLC for the corresponding film was provided.

The defect state density of films prepared by the in situ thermally dynamic crystallization process has been further characterized. The films were quantified for defect changes with and without the in situ thermally dynamic crystallization process, utilizing single-hole carrier devices. Single-hole carrier devices were prepared. The I–V curves in Figure 6 reveal two distinct regions, the ohmic and trap-filled limit regions. The defect density of states (Nt) for both holes and electrons in CsPbBr3 thin films was determined utilizing a single-hole carrier device. This was achieved through calculations performed as shown in Equation (3):

(3) $$N_{t}=\frac{2\varepsilon \varepsilon_{0}V_{\mathrm{TFL}}}{{\mathrm{eL}}^{2}}\;$$

where ε and ε_0_ represent the relative permittivity and vacuum permittivity of chalcogenide (8.8 × 10^−12^ F/m), respectively. VTFL corresponded to the defect limiting voltage, where the defective state was contained within this voltage range. L referred to the film thickness and was determined via a step meter test for the CsPbBr_3_ film. Finally, e represents the meta-charge. The thicknesses for the CsPbBr_3_ films at different temperatures could be found in the Supplementary Information, Table 1. The density of defects in chalcogenides prepared at room temperature (RT) and through in situ thermally dynamic crystallization at 40 °C is 8.9 × 10^13^ cm^−3^ and 1.6 × 10^13^ cm^−3^, respectively. Compared to RT, there is a notably reduced defect density at 40 °C.

By characterizing the light-emitting performances of CsPbBr_3_ PeLEDs prepared at different in situ thermally dynamic crystallization temperatures using the two-step spin-coating method, respectively, we can further analyze the effect of in situ thermally dynamic crystallization temperature on the properties of CsPbBr_3_ films.

**Figure 7:**
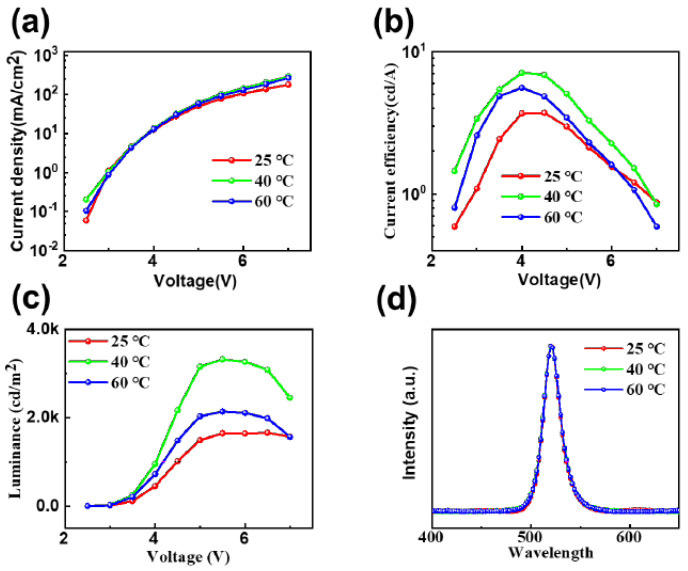
Lighting-emitting properties of CsPbBr_3_ PeLEDs prepared by the two-step method at RT, 40 °C and 60 °C in situ thermally dynamic crystallization temperatures. (**a**) Current–density voltage characteristic; (**b**) current efficiency voltage characteristic curve; (**c**) brightness voltage characteristic curve. (**d**) Normalized electroluminescence spectra of CsPbBr_3_ PeLEDs devices under 5 V driving voltage.

Figure 7a displays the current density–voltage curves of PeLEDs fabricated using the two-step method at room temperature, 40 °C, and 60 °C in situ heat-dynamic crystallization temperature. We discovered that at a temperature of 40 °C during in situ heat-dynamic crystallization and with the same operating voltage, the current density of each device was not significantly different. However, at lower voltages, the current density of the PeLEDs prepared under the conditions of RT and 60 °C differed due to the properties of their CsPbBr_3_ film compared to the current density of the CsPbBr_3_ film properties. The PeLEDs, which were prepared at room temperature and 60 degrees Celsius, exhibit reduced current densities as a result of the voids or clusters present in their CsPbBr_3_ films, leading to the preferential filling of defects by the carriers at lower voltages. The experiment also establishes that the CsPbBr_3_ films, processed through the low-temperature in situ thermally dynamic crystallization process at 40 °C, possess fewer defects, resulting in a lower proportion of nonradiative composites formed due to defect filling at a similar carrier injection rate. Consequently, the device achieves a higher current density for the same driving voltage. Figure 7b displays the current efficiency–voltage characteristic curves of PeLEDs at three temperatures: RT, 40 °C, and 60 °C. The figure indicates that the device’s current efficiency at 40 °C can soar up to 7.1 cd/A, which is almost two times higher than that of the PeLEDs without this process (RT, 3.6 cd/A). In Figure 7c, the device achieves a brightness of 3319 cd/m^2^ at a driving voltage of only 5 V and a temperature of 40 °C. This is in contrast to the brightness values of 1645 cd/m^2^ at room temperature (RT) and 2140 cd/m^2^ at higher in situ thermally dynamic crystallization temperatures. Figure 7d shows the CsPbBr_3_ Pe devices at RT, 40 °C, and 60 °C for in situ thermally dynamic crystallization. Meanwhile, Figure 7d illustrates the electroluminescence spectra of CsPbBr_3_ PeLEDs that were prepared at room temperature, 40 °C, and 60 °C using in situ thermally dynamic crystallization temperature. All of the spectra display identical luminescence peaks at 520 nm, which corresponds to the CIE color coordinates of (0.11, 0.77). It should be noted that the CIE color coordinates are also (0.11, 0.77). This suggests that the in situ crystallization process at low temperatures has little impact on the positions of the electroluminescence peaks in CsPbBr_3_ PeLEDs produced through the two-step method.

## 4. Conclusions

In this manuscript, vacuum thermal vapor deposition was utilized to deposit CsBr onto the surface of a PbBr_2_ film prepared via spin coating. A solid-state diffusion method (two-step process) was employed for the preparation of CsPbBr_3_ film, effectively overcoming the issue of cesium halide’s low solubility in organic solvents. Furthermore, the surface morphology of PbBr_2_ was regulated through in situ heat dynamic crystallization, leading to an increased specific surface area of the film and subsequently enhancing the solid-state diffusion rate. Based on these results, we fabricated electroluminescent devices using CsPbBr_3_ that achieved a maximum brightness of 3319 cd/m^2^ at 5 V along with a peak current efficiency of 7.1 cd/A. Furthermore, our in situ dynamic thermal crystallization process resulted in nearly a twofold increase in both brightness and current efficiency compared to the electroluminescent device without this process. In the solution method, the in situ thermal-assisted crystallization process is limited by solvent volatility. Although it can reduce the defect density of the thin film, its electroluminescence performance is still not high. In the vapor deposition method represented by vacuum thermal evaporation, the in situ assisted crystallization process can overcome the problem of solvent volatility and achieve a high-quality in situ crystallization of CsPbBr_3_ films, resulting in uniform grain size and low defect state density. Therefore, vacuum thermal evaporation plating technology based on in situ thermal-assisted crystallization can play a positive role in improving the performance of all inorganic perovskite electroluminescent devices represented by CsPbBr_3_.

## Figures and Tables

**Table 1:** The results of surface roughness and grain size of CsPbBr_3_ films prepared based on PbBr_2_ films deposited at room temperature, 40 °C, 60 °C and 80 °C in situ dynamic thermal crystallization temperatures.

	RT	40 °C	60 °C	80 °C
Sa (nm)	3	2	13	148
Sq (nm)	4	2	10	208
Size(nm)	289.6 ± 90.7	254.9 ± 36.9	236.0 ± 79.8	260.3 ± 135.2

**Table 2:** The width at half maximum of the diffraction peaks at the (020) and (040) crystal planes in PbBr_2_ films were measured. The thin films were prepared through in situ dynamic thermal crystallization at various temperatures.

	RT	40 °C	60 °C	80 °C
(020) FWHM	0.3°	0.18°	0.16°	0.12°
(040) FWHM	0.3°	0.26°	0.22°	0.22°

## Data Availability

The raw/processed data required to reproduce these findings cannot be shared at this time as the data also forms part of an ongoing study.

## Supplementary Materials

The following supporting information can be downloaded at: https://www.mdpi.com/article/10.3390/mi14112084/s1, Figure S1: (a–f) In situ assisted thermal crystallization temperatures under different cooling times, with an ambient temperature of 23 °C.
