# Supplementary material for: Perovskite Light-Emitting Devices Based on Solid-State Diffusion In Situ Dynamic Thermal Crystallization
Source: Micromachines (Basel). 2023 Nov 11;14(11):2084. doi: 10.3390/mi14112084 (PMC10673529; doi:10.3390/mi14112084)
Supplement: Supplementary file 1 [file micromachines-14-02084-s001.zip › micromachines-2670221-supplementary.pdf]

Support informations

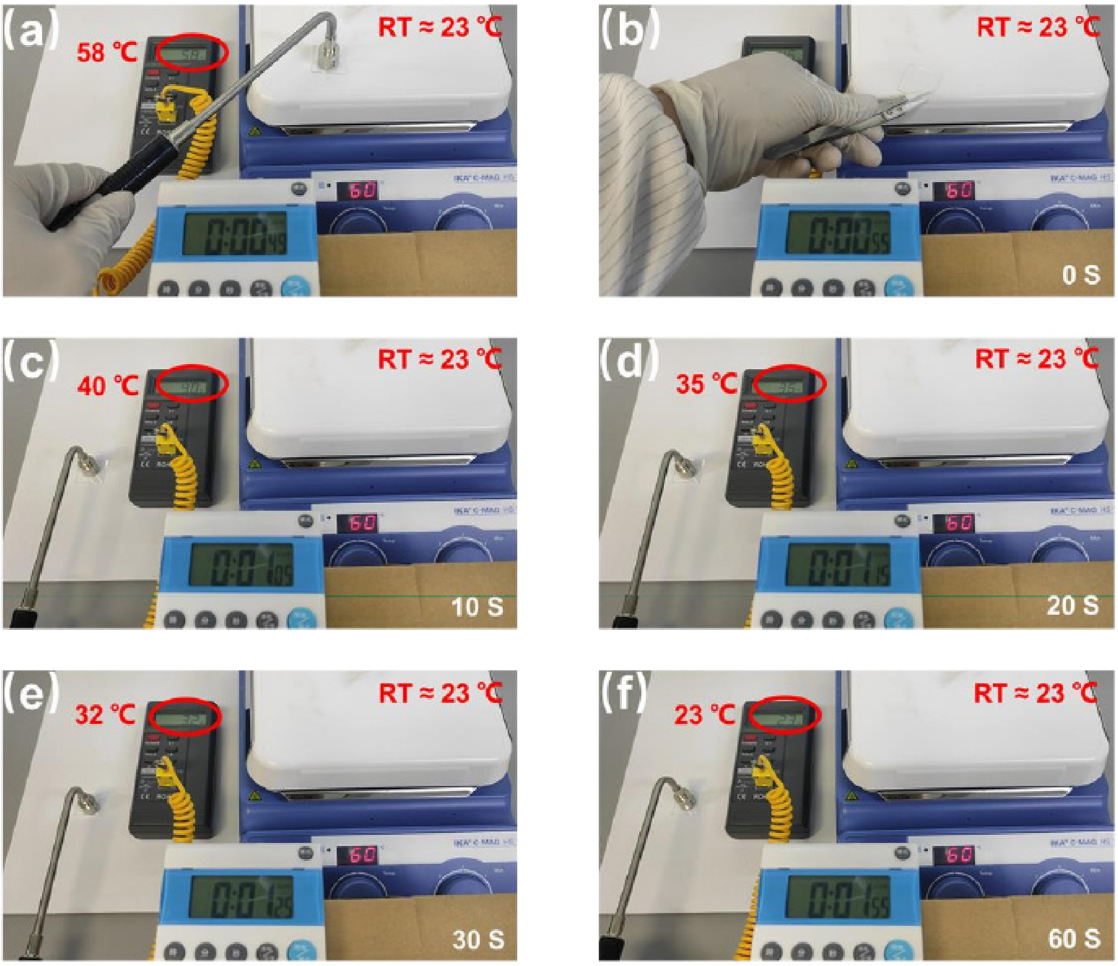

Figure S1. (**a**–**f**) In situ assisted thermal crystallization temperatures under different cooling times, with an ambient temperature of 23 ℃.
